# EZH2 inhibition induces senescence via ERK1/2 signaling pathway in multiple myeloma: EZH2 inhibition induces senescence in multiple myeloma

**DOI:** 10.3724/abbs.2024077

**Published:** 2024-05-27

**Authors:** Shushan Guo, Qiongwei Tang, Xuejie Gao, Liangning Hu, Ke Hu, Hui Zhang, Qikai Zhang, Yue Lai, Yujie Liu, Zhuning Wang, Shuaikang Chang, Yifei Zhang, Huifang Hu, Dong An, Yu Peng, Haiyan Cai, Jumei Shi

**Affiliations:** 1 Shanghai Clinical College Anhui Medical University Shanghai 200072 China; 2 Department of Hematology Shanghai East Hospital Tongji University School of Medicine Shanghai 200120 China; 3 The Fifth Clinical Medical College of Anhui Medical University Hefei 230022 China; 4 Department of Hematology Sir Run Run Shaw Hospital Zhejiang University Hangzhou 310016 China

**Keywords:** multiple myeloma, EZH2, Lamin B1, RRM2, cellular senescence

## Abstract

Epigenetic modifications play an important role in cellular senescence, and enhancer of zeste homolog 2 (EZH2) is a key methyltransferase involved in epigenetic remodeling in multiple myeloma (MM) cells. We have previously demonstrated that GSK126, a specific EZH2 inhibitor, exhibits anti-MM therapeutic efficacy and safety *in vivo* and *in vitro*; however, its specific mechanism remains unclear. This study shows that GSK126 induces cellular senescence in MM, which is characterized by the accumulation of senescence-associated heterochromatin foci (SAHF) and p21, and increased senescence-associated β galactosidase activity. Furthermore, EZH2 is inhibited in ribonucleotide reductase regulatory subunit M2 (RRM2)-overexpressing OCI-MY5 and RPMI-8226 cells. RRM2 overexpression inhibits the methyltransferase function of EZH2 and promotes its degradation through the ubiquitin-proteasome pathway, thereby inducing cellular senescence. In this senescence model, Lamin B1, a key component of the nuclear envelope and a marker of senescence, does not decrease but instead undergoes aberrant accumulation. Meanwhile, phosphorylation of extracellular signal-regulated protein kinase (ERK1/2) is significantly increased. The inhibition of ERK1/2 phosphorylation in turn partially restores Lamin B1 level and alleviates senescence. These findings suggest that EZH2 inhibition increases Lamin B1 level and induces senescence by promoting ERK1/2 phosphorylation. These data indicate that EZH2 plays an important role in MM cellular senescence and provide insights into the relationships among Lamin B1, p-ERK1/2, and cellular senescence.

## Introduction

Cellular senescence is a stable state of cell cycle arrest and an important mechanism for preventing tumor cell proliferation [ 1‒ 3] . Cellular senescence is associated with haematopoietic stem cell function and the progression of multiple myeloma (MM) [ 4‒ 6] . The senescence response has been reported to be a key factor affecting chemotherapy sensitivity and treatment outcomes in patients with haematological malignancies. For example, AKI603 overcomes imatinib resistance in chronic myeloid leukaemia cells by inducing senescence [7]. Thus, unravelling the mechanisms of cellular senescence in MM may be key to overcoming therapeutic barriers in patients with relapsed/refractory MM.

Recently, several studies have focused on oncogene-induced senescence (OIS) [ 8‒ 10] . OIS is a persistent antiproliferative response that acts as a barrier against malignant transformation. Senescence-associated heterochromatin foci (SAHF) are multi-layered structures centered on a condensed heterochromatic core that can be detected as DNA/chromatin-dense foci and can be induced by various trigger factors. Usually, OIS is accompanied by SAHF, whereas other forms of DNA damage response-induced senescence exhibit less SAHF [11]. Mitochondria dysfunction is also a representative hallmark of senescence [ 11‒ 13] . Normal cristae are crucial for mitochondrial function [14], while mitochondria from old samples lose their typical crista structure [15].

Enhancer of zeste homolog 2 (EZH2), a catalytic subunit of polycomb repressive complex 2 (PRC2), plays a key role in the epigenetic remodeling of MM through the trimethylation of Lys27 in histone 3 (H3K27me3) [ 16‒ 18] . Recent studies have shown that epigenetic modification plays an important role in cellular senescence [11]. In addition, inhibitors targeting EZH2 have recently been demonstrated to have therapeutic efficacy against various tumors [ 19, 20] . High expression of EZH2 is associated with poor outcome in hematologic malignancies (myeloma and chronic lymphocytic leukaemia). In a previous study, we demonstrated that GSK126, a specific EZH2 inhibitor, exhibits anti-MM therapeutic efficacy and safety *in vivo* and *in vitro* [21]; however, its specific mechanism remains unclear. EZH2 plays a critical role in normal B cell development, and its expression level influences differentiation decisions [22]. EZH2 is degraded via the ubiquitin-proteasome pathway [23]. Phosphorylated EZH2 acts as a tumor suppressor, resulting in the inhibition of its own methyltransferase function [ 24, 25] . Several studies have shown that EZH2 is involved in maintaining cell stemness as well as cellular senescence in several cancers [ 26‒ 28] . Furthermore, EZH2 depletion activates p21 and induces senescence in melanoma [ 29, 30] . However, the relationship between EZH2 and senescence in MM remains unclear. ribonucleotide reductase regulatory subunit M2 (RRM2) is a major chromosomal instability gene associated with the cell cycle [ 31‒ 33] . Recent studies suggested that RRM2 may play an important role as a bridge between senescence and cancer [ 34, 35] . However, whether the role of EZH2 in senescence is influenced by RRM2 has never been reported.

Lamin B1 is the primary structural component of the nuclear envelope. The nuclear lamina is closely attached to the inner membrane of the nuclear envelope and contributes to the size, shape, and mechanical stability of the nucleus. Changes in Lamin B1 level affect cellular senescence [ 36, 37] . Lamin B1 deficiency is a common marker of cellular senescence [ 11, 38] . The distribution of Lamin B1 is closely related to SAHF formation during senescence [39]. Extracellular signal-regulated protein kinase (ERK1/2) is a mitogen-activated protein kinase with typical cascade signaling properties that plays a key role in signal transduction pathways. In most cases, ERK1/2 is a regulator of cell proliferation; however, it can also promote senescence [ 40‒ 42] .

Recently, a series of therapeutic strategies targeting cellular senescence have been developed, including the induction of cellular senescence and elimination of senescent cells [ 41, 43] . In the present study, we focused on the role of EZH2 in the induction of senescence in MM cells. Our findings may initiate further research on drug resistance mechanisms and the establishment of senescence-related therapeutic strategies for patients with MM.

## Materials and Methods

### Reagents and antibodies

GSK126 (S7061) was obtained from Selleck Chemicals (Shanghai, China). Chloroquine (T8689) was obtained from TargetMol (Boston, USA). MG132 (HY-13259), AG126 (HY-108330) and cycloheximide (HY-12320) were purchased from MedChemExpress (Monmouth Junction, USA). Anti-RRM2 antibody (ab172476) was purchased from Abcam (Cambridge, USA). Anti-p21 (2947T), p-ERK1/2 (9101S), phospho-Rb (Ser780) (9307S), p44/42 MAPK (ERK1/2) (9102S), and EZH2 (5246T) antibodies were purchased from Cell Signaling Technology (Danvers, USA). Anti-GAPDH (60004-1-Ig), Histone H3K27me3 (39157), and Lamin B1 (66095-1-Ig) antibodies were purchased from Proteintech (Wuhan, China). Anti-ubiquitin antibody (A19686) was purchased from ABclonal (Wuhan, China). DAPI was purchased from Beyotime (Shanghai, China).

### Cells and cell culture

OCI-MY5 and RPMI-8226 cell lines were purchased from the American Type Culture Collection (Manassas, USA) or obtained as previously described [44]. Cells were cultured in RPMI 1640 medium (Gibco, Waltham, USA) supplemented with 10% fetal bovine serum.

### Western blot analysis

Western blot analysis were performed as described previously [45]. Briefly, cells (3×10 ^5^ cells/mL) were treated with GSK126 for the indicated time periods, collected, and then lysed with lysis buffer. Subsequently, the proteins were subjected to sodium dodecyl sulfate‒polyacrylamide gel electrophoresis (SDS-PAGE) and transferred onto nitrocellulose membranes. The membranes were blocked and then incubated with anti-p21, p-ERK1/2, phospho-Rb (Ser780), p44/42 MAPK (ERK1/2), EZH2, H3K27me3, and Lamin B1 antibodies. After incubation with the corresponding HRP-conjugated secondary antibody, Immobilon Western Chemiluminescent HRP Substrate (WBKLS0500; Millipore, Darmstadt, Germany) was used for detection using an Odyssey infrared imaging system (LI-COR Biosciences, Lincoln, USA).

### Immunofluorescence assay

Immunofluorescence assays were performed as described previously [45]. Briefly, the cells were collected and fixed with 4% paraformaldehyde (Sigma-Aldrich, Darmstadt, Germany) for 15 min. The cells were permeabilized with 0.1% Triton X-100 and blocked with 0.3% bovine serum albumin. Anti-EZH2 and Lamin B1 antibodies were added to the glass slides and incubated. After staining with 4′,6-diamidino-2-phenylindole (DAPI; Beyotime), images were captured under a confocal laser scanning microscope (Carl Zeiss, Oberkochen, Germany). The images were analyzed using Fiji ImageJ 2.1.0 software (National Institute of Health, Bethesda, USA).

### Co-immunoprecipitation (Co-IP) assay

Co-IP was performed as described previously [45]. Briefly, cells were lysed in cell lysis buffer (P0013; Beyotime) containing protease inhibitor cocktails and phenylmethyl sulfonyl fluoride for 40 min at 4°C. The supernatant was incubated with a specific antibody and protein A/G agarose resin (36403ES08; Yeasen, Shanghai, China). The beads were then washed with lysis buffer, proteins were eluted by boiling for 5 min in SDS-PAGE sample loading buffer, and western blot analysis was performed.

### Construction of stable expression cell lines

OCI-MY5 and RPMI-8226 cells were infected with RRM2-overexpressing (OE) or empty-vector (EV) lentiviruses in HiTransG P infection-enhancing solution (1×) and incubated for 20 h. RRM2 OE and EV lentiviruses were purchased from Jikai Gene (Shanghai, China). Stable cells were screened with 3 μg/mL puromycin for 7 days according to the manufacturer’s instructions.

### Senescence-associated β-galactosidase (SA-β-gal) staining analysis

SA-β-gal staining was performed using an SA-β-gal staining kit (C0602; Beyotime). MM cells were seeded in 12-well plates. After treatment, the cells were collected and rinsed with phosphate-buffered saline. A fixative (1 mL) was added to fix the cells. Then cells were washed and coincubated with a working solution of β-gal and X-Gal at 37°C overnight. Positively stained cells were imaged by light microscopy and counted in three random fields.

### Transmission electron microscopy

RPMI-8226 cells were pretreated with the indicated drugs, collected by centrifugation, fixed with 2.5% glutaraldehyde, and stored at 4°C. The cells were then rinsed three times with 0.1 M sulfuric acid buffer (pH 7.4) for 15 min each time, fixed with 1% osmic acid+ 0.1 M sulfuric acid buffer (pH 7.4) at 20°C for 2 h, and rinsed three times with 0.1 M phosphate buffer. The cells were then dehydrated by 30%, 50%, 70%, 80%, 85%, 90%, and 100% (twice) alcohol for 15‒20 min each, and infiltrated with acetone and epoxy resin. After embedding, 80‒100 nm sections were prepared with an ultrathin slicer. Uranium-lead double staining (2% acidic uranium saturated aqueous solution, lead citrate) was performed for 15 min at room temperature. A Tecnai G20 TWIN transmission electron microscope (FEI, Eindhoven, Netherlands) was used for visual analysis.

### Statistical analysis

Statistical analyses were performed using GraphPad Prism 8.0.1 (GraphPad Software, San Diego, USA). Data are presented as the mean±SD. Student’s *t* test was used to determine the significance of differences between two groups. Statistical significance was set at *P*<0.05.

## Results

### The EZH2-specific methyltransferase inhibitor GSK126 induces cellular senescence in MM cells

**Figure 1:**
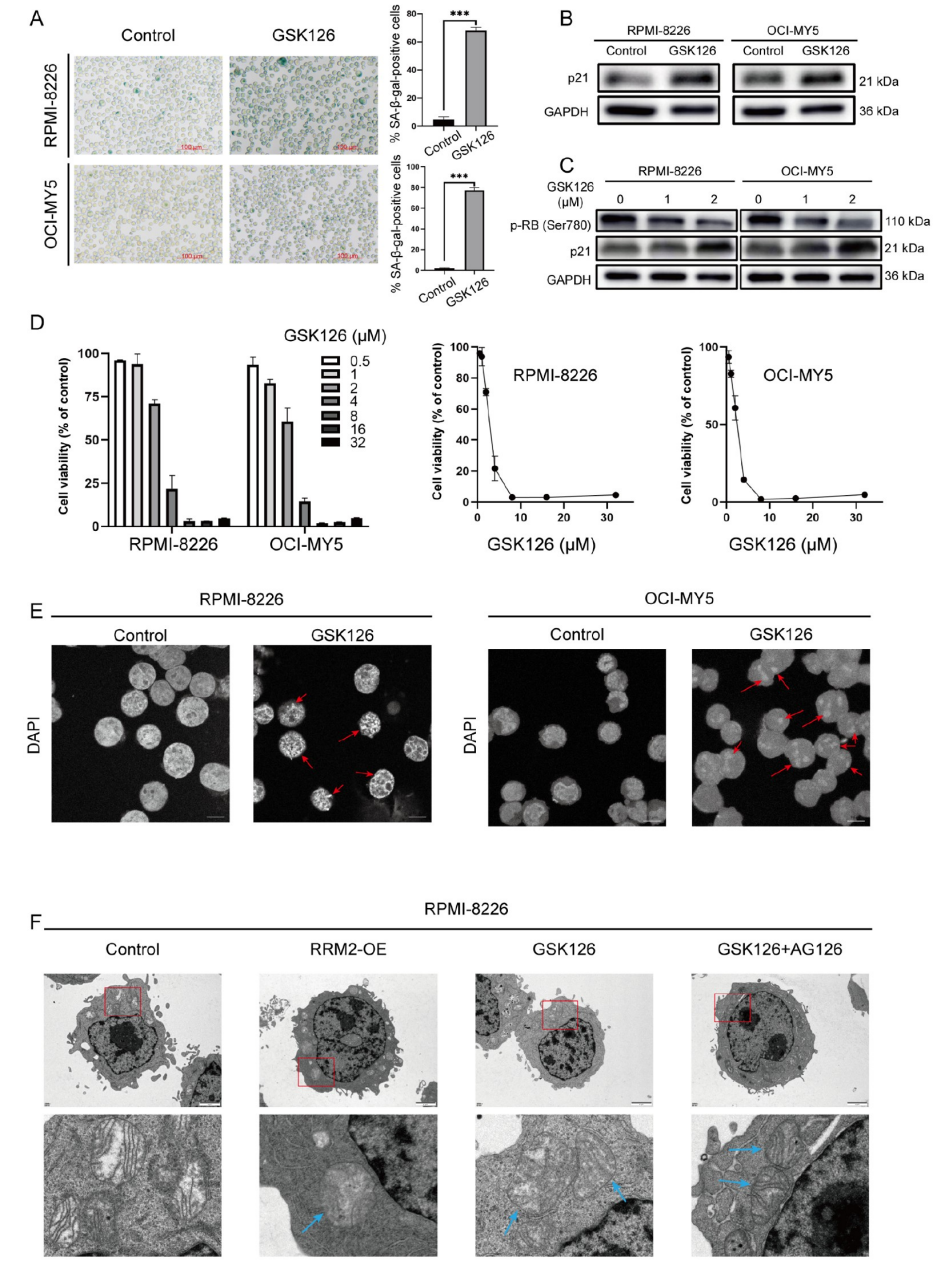
Figure 1 The EZH2-specific methyltransferase inhibitor GSK126 induced cellular senescence in MM (A) SA-β-gal expression in GSK126-treated and control RPMI-8226 and OCI-MY5 cells. Representative photomicrographs at the same magnification are shown. Scale bar: 100 μm. Quantification of SA-β-gal expression are shown. Data are presented as the mean±SD. (B) Western blots showing p21 levels in RPMI-8226 and OCI-MY5 cells after GSK126 treatment. (C) Western blots showing p21 and p-RB (Ser780) levels in RPMI-8226 and OCI-MY5 cells after GSK126 treatment (0, 1, and 2 μM). (D) MM cells were treated with GSK126 for 24 h, and cell viability was determined by CCK-8 assay. (E) RPMI-8226 and OCI-MY5 cells were examined by immunofluorescence with DAPI (blue). Using Fiji ImageJ 2.1.0 software, we split the color via the split channel and analyzed the images in 8-bit grayscale on the blue channel. Red arrows show cells with SAHF. Scale bar: 10 μm. (F) Representative images of chromatin repositioning and mitochondrial morphology in senescent RPMI-8226 cells. Control cells showed largely homogeneous chromatin organization (with slightly enhanced staining around the inner nuclear envelope and nucleoli), and RRM2 OE or GSK126 treatment resulted in the formation of multiple patchy dense heterochromatin structure domains in the nucleus. Meanwhile, in the RRM2 OE or GSK126-treated cells, blue arrows indicate swollen mitochondria lacking cristae. Addition of AG126 partially restored the global distribution of chromatin and repaired mitochondria. Scale bar: 2 μm. The bottom row contains images enlarged from the boxed area in the corresponding panel in the top row. *** P<0.001.

Our previous study demonstrated the therapeutic effect of GSK126 on MM both *in vivo* and *in vitro* [21]. In this study, we investigated the effects of an EZH2 inhibitor (GSK126) on senescence in OCI-MY5 and RPMI-8226 cells. SA-β-gal assay showed that the GSK126-treated group was characterized by a higher proportion of senescent cells ( Figure 1A). The accumulation of p21 is a hallmark of cellular senescence. The results showed that compared with the control group, GSK126-treated cells had a higher level of p21 ( Figure 1B). GSK126 promotes an increase in p21 and a decrease in p-RB in a concentration-dependent manner ( Figure 1C). The half-maximal inhibitory concentration (IC _50_) values of GSK126 for these cell lines were 2.648 μM for RPMI-8226 cells and 2.19 μM for OCI-MY5 cells ( Figure 1D). Furthermore, following the reported experimental method [ 8, 46, 47] , we used DAPI staining to observe nuclear chromatin changes in OCI-MY5 and RPMI-8226 cells. We observed that GSK126 treatment resulted in the “aggregation-densification” of DAPI staining under confocal microscopy, which is indicative of SAHF ( Figure 1E) and is usually associated with OIS. To make the results easier to observe, we decolorized the images using ImageJ software. Red arrows indicate the cells with SAHF ( Figure 1E). To further confirm cellular senescence, we used TEM. Figure 1F shows transmission electron microscopy images of chromatin and mitochondrial morphology. In control RPMI-8226 cells, there was largely homogeneous chromatin organization and normal mitochondria with intact cristae, while in GSK126-treated cells, multiple patchy dense heterochromatin structure domains formed in the nucleus. In addition, blue arrows indicate swollen mitochondria lacking cristae. These results demonstrated that the EZH2-specific methyltransferase inhibitor GSK126 induced senescence in MM cells.

### Overexpression of RRM2 inhibits EZH2 methyltransferase function and induces senescence in MM cells

**Figure 2:**
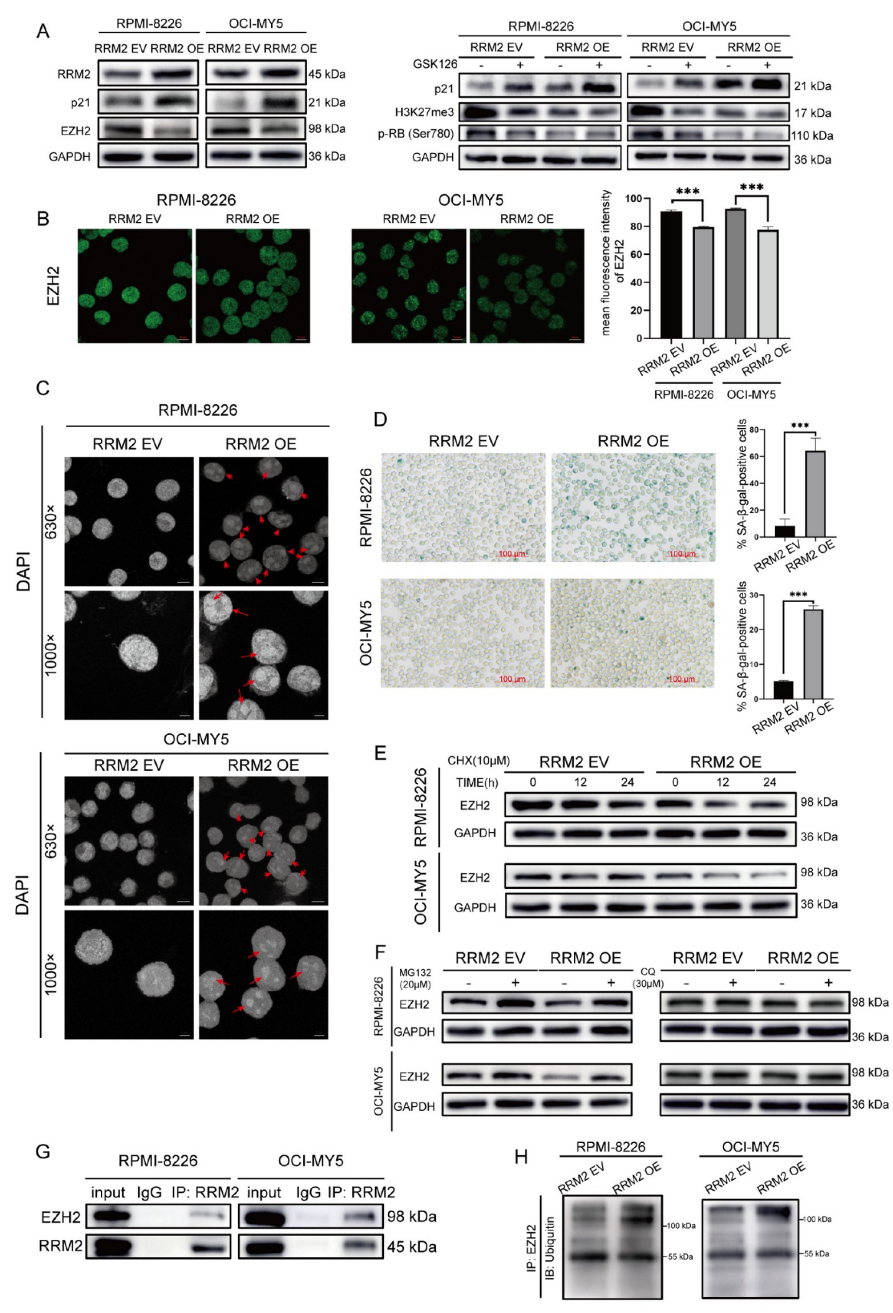
Figure 2 Overexpression of RRM2 inhibited EZH2 methyltransferase activity and induced senescence in MM cells (A) Western blots showing RRM2, EZH2, H3K27me3, p-RB (Ser780), and p21 levels in RPMI-8226 and OCI-MY5 cells after infected with RRM2 OE or EV lentiviruses in the presence or absence of GSK126 (1.5 μM for 24 h). GAPDH was used as a loading control. (B) Immunofluorescence staining of EZH2 (green) in MM cells. Scale bar: 10 μm. Right panel: The mean fluorescence intensity of EZH2 (green) was lower in RRM2 OE cells than in RRM2 EV cells. (C) RRM2 EV and RRM2 OE cells were stained with DAPI (blue). The photomicrographs were split via the split channel and analyzed in an 8-bit grayscale on the blue channel. Red arrows show representative cells with SAHF. Scale bar: 5 μm or 10 μm. (D) SA-β-gal staining of RRM2 OE or EV cells. Representative photographs are shown. Scale bar: 100 μm. Quantification of SA-β-gal expression is shown. (E) Cells were treated with 10 μM CHX for 0, 12, or 24 h. Protein expression level of EZH2 was measured by western blot analysis. GAPDH was used as a loading control. (F) Cells were treated with MG132 (20 μM for 12 h) or CQ (30 μM for 4 h). EZH2 level was measured by western blot analysis. (G) Total protein lysates of RPMI-8226 and OCI-MY5 cells were collected for co-immunoprecipitation using anti-RRM2 and anti-EZH2 antibodies. (H) Cell lysates were subjected to immunoprecipitation with anti-EZH2 antibody, followed by western blot analysis with an anti-ubiquitin antibody. *** P<0.001.

We wondered whether the role of EZH2 in senescence is affected by RRM2. In fact, EZH2 alters the expression of its downstream target genes through H3K27me3. Western blot analysis results showed that EZH2 and H3K27me3 levels decreased in RRM2 OE cells ( Figure 2A). Immunofluorescence microscopy analysis was also performed to detect EZH2. The mean fluorescence intensity of EZH2 (green) was lower in RRM2 OE cells than in RRM2 EV cells ( Figure 2B). Then, we examined the effects of RRM2 overexpression on senescence in MM cells. Interestingly, RRM2 overexpression resulted in an increase in the number of SA-β-gal-positive cells ( Figure 2D), the accumulation of p21 ( Figure 2A), and the formation of SAHF ( Figure 2C). Either GSK126 treatment or RRM2 overexpression resulted in an increase in p21 and a decrease in p-RB level, and simultaneous addition of GSK126 and RRM2 overexpression resulted in a further increase in p21 and a further decrease in p-RB level ( Figure 2A). Moreover, as shown in Figure 1F, RRM2 OE cells exhibited chromatin repositioning and swollen mitochondria lacking cristae (blue arrows). Taken together, these data show that RRM2 overexpression inhibits EZH2 methyltransferase activity and induces senescence in MM cells.

Furthermore, we explored the mechanism by which RRM2 inhibits EZH2. After treatment with cycloheximide (CHX) to inhibit protein synthesis, EZH2 was degraded faster in RRM2 OE cells than in RRM2 EV cells, indicating that RRM2 promoted EZH2 degradation ( Figure 2E). Most cellular proteins are degraded by the ubiquitin-proteasome system or the autophagy-lysosomal pathway. RPMI-8226 and OCI-MY5 cells were treated with a proteasome inhibitor (MG132) or an autophagy inhibitor (chloroquine, CQ). EZH2 reduction was significantly mitigated by treatment with the proteasome inhibitor MG132 ( Figure 2F). Additionally, the autophagy inhibitor CQ could not mitigate the reduction in EZH2 induced by RRM2 ( Figure 2F). Co-immunoprecipitation results showed that RRM2 bound to EZH2 ( Figure 2G). In addition, EZH2 ubiquitination level was increased in RRM2 OE cells compared to that in RRM2 EV cells ( Figure 2H). These results revealed that RRM2 promotes EZH2 degradation via the ubiquitin-proteasome pathway.

### EZH2 inhibition induces aberrant upregulation of Lamin B1 in senescent MM cells

**Figure 3:**
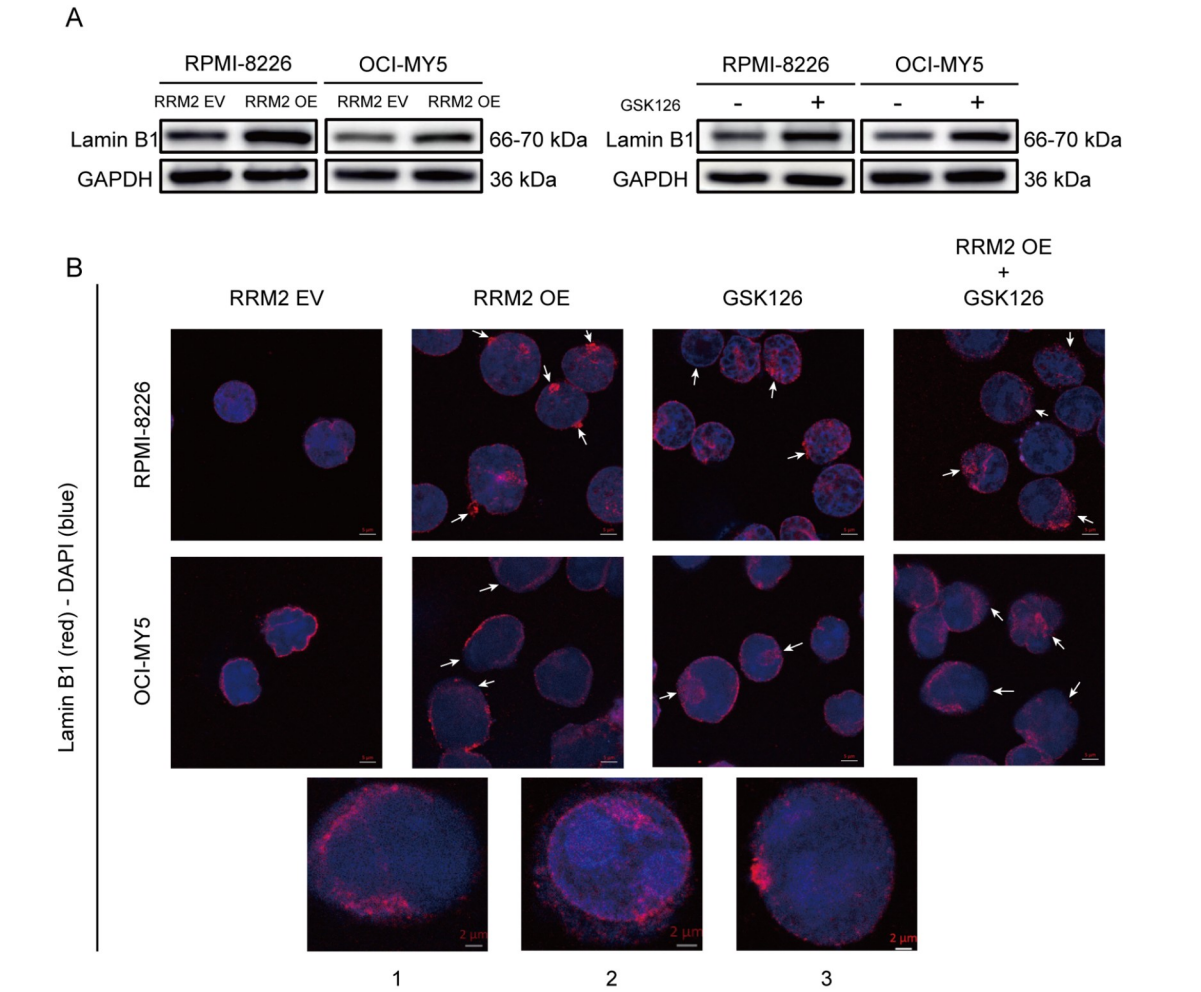
Figure 3 The abnormality of Lamin B1 in MM senescent cells (A) Lamin B1 protein level was measured by western blot analysis in RRM2 OE- or GSK126 (1.5 μM for 24 h)-treated MM cells. (B) RRM2 EV and RRM2 OE cells, GSK126-treated or untreated, were subjected to immunofluorescence staining with anti-Lamin B1 antibody (red) and DAPI staining (blue). Scale bar: 5 μm. Absence of Lamin B1 in the nuclear envelope (1), diffused Lamin B1 around the nuclear envelope (2), and perinuclear Lamin B1 aggregates (3) were observed in senescent MM cells. Scale bar: 2 μm.

Both the addition of GSK126 and RRM2 overexpression increased Lamin B1 level ( Figure 3A). We speculate that the increase in Lamin B1 level is involved in the senescence of MM cells induced by EZH2 inhibition. Immunofluorescence staining of Lamin B1 and DAPI staining were performed and observed under a confocal microscope ( Figure 3B). In control MM cells, Lamin B1 was uniformly expressed in the inner nuclear envelope, which appeared as a clear and thin membrane. However, the distribution of Lamin B1 in the nuclear envelope was abnormal in RRM2 OE- or GSK126-treated cells, which was characterized by (1) partial absence in the inner nuclear envelope; (2) diffusion within the nucleus or along the periphery of the nuclear envelope without a discrete boundary; and (3) an increase in perinuclear Lamin B1 aggregates. These data indicate that EZH2 inhibition induces aberrant upregulation and distribution of Lamin B1 in senescent MM cells.

### Increased phosphorylation of ERK1/2 is required for cellular senescence induced by the inhibition of EZH2

**Figure 4:**
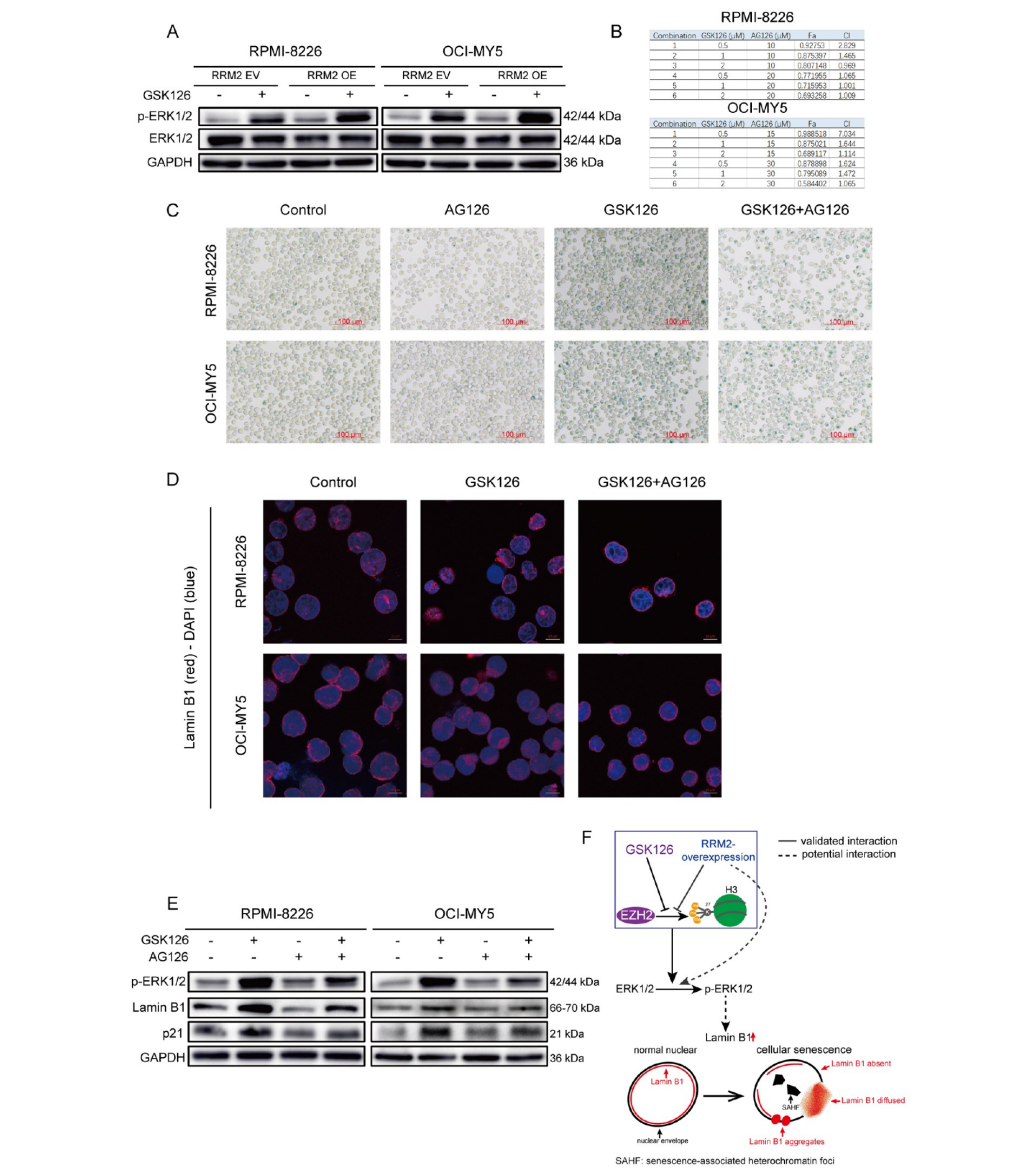
Figure 4 Increased phosphorylation of ERK1/2 is required for EZH2 inhibition-induced cellular senescence (A) p-ERK1/2 and ERK1/2 protein levels were measured by western blot analysis in RRM2 OE MM cells in the presence or absence of GSK126 (1.5 μM– 24 h). (B) RPMI-8226 and OCI-MY5 cells were treated with various concentrations of GSK126 and AG126 for 24 h. Cell viability was measured by CCK-8 assay. CI values were calculated using the CalcuSyn software. A CI>1 indicated antagonism between GSK126 and AG126. (C) RPMI-8226 and OCI-MY5 cells were pre-treated with GSK126 (1.5 μM) for 2 h and then cocultured with AG126 (30 μM) for 22 h. SA-β-gal activity was measured. Scale bar: 100 μm. (D) Similar to the treatment in (C). Cells were then examined by immunofluorescence staining with anti-Lamin B1 antibody (red) and DAPI staining (blue). Scale bar: 10 μm. In the control group, Lamin B1 was uniformly expressed in the inner nuclear envelope, whereas the distribution of Lamin B1 in the nuclear envelope was abnormal after GSK126 treatment. Meanwhile, the addition of AG126 partially restored the uniform distribution of Lamin B1 in the nuclear envelope. (E) Similar to the treatment in (C). Cells were collected, and the cell lysates were subjected to western blot analysis with the indicated antibodies. (F) A model depicting the key molecular mechanisms of cellular senescence induced by EZH2 inhibition in MM. First, GSK126, a methyltransferase inhibitor of EZH2, induces cellular senescence by promoting ERK1/2 phosphorylation. Second, RRM2 overexpression downregulates EZH2 and inhibits its methyltransferase function. In addition, RRM2 overexpression promotes ERK1/2 phosphorylation and induces cellular senescence. These senescent MM cells are characterized by SAHF formation and abnormal Lamin B1 distribution. The solid lines indicate direct interactions, while the dashed lines indicate potential interactions.

ERK1/2 is a key messenger of extracellular and intracellular signals that plays a crucial role in cell proliferation, differentiation, and cellular senescence [48]. Abnormally high ERK1/2 activation triggers OIS [ 49, 50] , thereby preventing tumor progression [51]. Both the addition of GSK126 and RRM2 overexpression promoted a significant increase in p-ERK1/2 levels ( Figure 4A). Therefore, we explored whether p-ERK1/2 is involved in EZH2-induced senescence in MM cells. We then used AG126, a specific phosphorylation inhibitor of ERK1/2. GSK126 and AG126 exhibited antagonistic effects mainly on RPMI-8226 and OCI-MY5 cells ( Figure 4B). The SA-β-gal assay results showed that AG126 mitigated EZH2-induced cellular senescence ( Figure 4C). The addition of AG126 restored the uniform distribution of Lamin B1 in the inner nuclear envelope ( Figure 4D). Western blot analysis revealed that AG126 also mitigated the EZH2-induced aberrant upregulation of Lamin B1 and p21 while reducing p-ERK1/2 ( Figure 4E). As shown in Figure 1F, RRM2 OE- or GSK126-treated cells were characterized by swollen mitochondria lacking cristae. Subsequently, when AG126 was added, we observed the recovery of mitochondria and their cristae. In summary, increased phosphorylation of ERK1/2 is required for cellular senescence induced by the inhibition of EZH2 in MM cells.

## Discussion

Targeting EZH2 has exhibited therapeutic effects in various haematological malignancies [ 19, 52, 53] . In our previous study, we demonstrated that GSK126, an EZH2 inhibitor, exhibited therapeutic efficacy and safety in MM xenograft mice [21], but its specific mechanism remains unclear. EZH2 depletion activates p21 and induces senescence in melanoma [ 29, 30] . P21 has been described as a marker of cellular senescence, and p-RB depletion implies cell cycle arrest [54]. In the present study, we found that EZH2 inhibition activated p21, decreased p-RB, promoted the accumulation of Lamin B1, and promoted SAHF formation in MM cells. The SAHF is a multi-layered structure centered on a condensed heterochromatic core. SAHF can serve as an important barrier to cell fate transitions and enhance cell fate determination. Studies have shown that mitochondrial dysfunction is a general feature of cellular senescence [ 11, 12, 55‒ 57] . We observed abnormal mitochondrial morphology in the senescent MM cells, such as swollen mitochondria and damaged mitochondrial cristae compared to controls, which indicates mitochondrial dysfunction. Therefore, EZH2 inhibition exerts an anti-MM effect by affecting cellular senescence.

Furthermore, RRM2 may play an important role as a bridge between senescence and cancer development [ 34, 35] . We found that EZH2 was inhibited in RRM2 OE cells. EZH2 is a histone methyltransferase involved in the development of MM by trimethylating histone H3 at Lys27, thereby silencing target genes. RRM2 overexpression not only reduced the protein level of EZH2 to some extent but also effectively inhibited the ability of EZH2 to catalyze H3K27me3 ( Figure 2A). Similarly, GSK126, a methyltransferase inhibitor of EZH2, primarily inhibits the ability of EZH2 to catalyze H3K27me3 ( Figure 2A). The simultaneous addition of GSK126 and RRM2 overexpression resulted in a further increase in p21 and a further decrease in H3K27me3 level, suggesting that this is a major mechanism underlying cellular senescence. Downregulation of EZH2 expression in RRM2 OE cells was mitigated by MG132, indicating that EZH2 was degraded faster through the ubiquitin‒proteasome system in RRM2 OE cells than in RRM2 EV cells. In this study, we found that RRM2 overexpression inhibited EZH2 and induced cellular senescence simultaneously. However, further studies are needed to investigate whether RRM2 OE induces cellular senescence solely through the EZH2 pathway. After overexpression of RRM2, we observed a relatively slight decrease in EZH2 protein level but a significant decrease in H3K27me3 level. We therefore hypothesized that RRM2 may regulate the methyltransferase function of EZH2 in other ways in addition to reducing EZH2 protein level. Phosphorylation of EZH2 has been reported to regulate its own methyltransferase function, so whether RRM2 regulates other post-translational modifications of EZH2, such as phosphorylation, needs to be further investigated.

The distribution of Lamin B1 is closely related to SAHF formation during senescence [39]. Lamin B1 deficiency is a common marker of cellular senescence [ 11, 38] . We observed abnormal upregulation of Lamin B1 in senescent MM cells, providing novel insights into its role in cellular senescence. High level of Lamin B1 in Ataxia telangiectasia cells results in altered nuclei and senescence [8], which is parallel to what we have demonstrated in OCI-MY5 and RPMI-8226 cells. Therefore, we hypothesized that the homeostasis of Lamin B1 protein level is essential for maintaining normal cellular function. This finding suggested that tight regulation of Lamin B1 level is essential, as both its deficiency and excess can regulate cellular senescence.

Studies have suggested that ERK1/2 promotes cellular senescence [ 49, 50] . In this work, GSK126 (an EZH2 inhibitor) induced cellular senescence by promoting ERK1/2 phosphorylation, while RRM2 overexpression also inhibited EZH2 and promoted ERK1/2 phosphorylation and cellular senescence. A specific inhibitor of ERK1/2 phosphorylation (AG126) partially alleviated the abnormal expression of Lamin B1 and alleviated the cellular senescence induced by GSK126 ( Figure 4B‒E). The addition of AG126 partially restored the global distribution of chromatin and repaired mitochondria, which was visualized by TEM ( Figure 1F). This finding provides new evidence for the involvement of ERK1/2 in the regulation of cellular senescence. However, how EZH2 inhibition promotes ERK1/2 phosphorylation needs to be further investigated. In addition, whether EZH2 is the only downstream pathway of RRM2 to promote ERK1/2 phosphorylation is also a question that needs to be explored.

Recently, a range of therapeutic strategies have been developed to target cellular senescence, including the induction of cellular senescence and elimination of senescent cells [58]. Haematopoietic stem cell transplantation and chimeric antigen receptor-T cell therapy are two key approaches for treating MM. The regulation of senescence in haematopoietic stem cells is associated with their therapeutic efficacy. Additionally, targeting senescent cells may help to overcome chemoresistance. Our findings provide valuable insights for the optimization of therapeutic regimens for haematological malignancies.
